# Effects of 5 MeV Proton Irradiation on Nitrided SiO_2_/4H-SiC MOS Capacitors and the Related Mechanisms

**DOI:** 10.3390/nano10071332

**Published:** 2020-07-08

**Authors:** Dongxun Li, Yuming Zhang, Xiaoyan Tang, Yanjing He, Hao Yuan, Yifan Jia, Qingwen Song, Ming Zhang, Yimen Zhang

**Affiliations:** 1 School of Microelectronics, Xidian University, The State Key Discipline Laboratory of Wide Band Gap Semiconductor Technology, Xi’an 710071, China; lidxun@126.com (D.L.); zhangym@xidian.edu.cn (Y.Z.); haoyuan@xidian.edu.cn (H.Y.); qwsong@xidian.edu.cn (Q.S.); ymzhang@xidian.edu.cn (Y.Z.); 2 School of Electronic Engineering, Xi’an University of Posts and Telecommunications, Xi’an 710121, China; jiayifan@xupt.edu.cn; 3 Beijing Institute of Spacecraft System Engineering, Beijing 100094, China; 13426209018@139.com

**Keywords:** SiO_2_/4H-SiC MOS capacitors, proton irradiation, ionization effect, displacement effect, interface traps, near interface traps, flat-band voltage

## Abstract

In this paper the effects of 5 MeV proton irradiation on nitrided SiO_2_/4H-SiC metal–oxide–semiconductor (MOS) capacitors are studied in detail and the related mechanisms are revealed. The density of interface states (Dit) is increased with the irradiation doses, and the annealing response suggests that the worse of Dit is mainly caused by displacement effect of proton irradiation. However, the X-rays photoelectron spectroscopy (XPS) measurement shows that the quantity proportion of breaking of Si≡N induced by displacement is only 8%, which means that the numbers of near interface electron traps (NIETs) and near interface hole traps (NIHTs) are not significantly changed by the displacement effect. The measurements of bidirectional high frequency (HF) C-V characteristics and positive bias stress stability show that the number of un-trapped NIETs and oxide electron traps decreased with increasing irradiation doses because they are filled by electrons resulted from the ionization effect of proton irradiation, benefiting to the field effective mobility (μ_FE_) and threshold voltage stability of metal–oxide–semiconductor field-effect transistors (MOSFETs). The obviously negative shift of flat-band voltage (V_FB_) resulted from the dominant NIHTs induced by nitrogen passivation capture more holes produced by ionization effect, which has been revealed by the experimental samples with different nitrogen content under same irradiation dose.

## 1. Introduction

Because of the excellent electrical and thermal properties, silicon carbide (SiC) is a promising wide-bandgap semiconductor material with application in the power, high temperature, and space electronic fields [1,2]. With the improvement of the μ_FE_ caused by the nitrogen passivation of SiC/SiO_2_ interface, high voltage power metal–oxide–semiconductor field-effect transistor (MOSFET) switches have been available in the commercial market since 2011. SiC-based power devices are expected to be used in aerospace domain for substantially saving weight and enhancing performance. But the different types of irradiations in space environment will change the device electrical characteristics or even lead to the permanent failures of devices. The SiC devices are hopeful to exhibit high radiation hardness due to its high displacement threshold energy and the wide bandgap. Therefore, more research interests are turned to the irradiation effect of the nitrided MOS capacitors and power MOSFETs. The previous studies focused on the total dose radiation response of γ-ray and X-ray, which are only considered as ionization effect [3,4,5,6,7,8,9,10]. The negative shift of V_FB_ and threshold voltage (V_TH_) is the major problem due to the positive charges generated in the gate oxide during irradiation, but the mechanisms of formation of positive charges are required to be further studied. In addition, the effect of ionization irradiation on near interface traps which can significantly influence the μ_FE_ of MOSFET has not been investigated.

Also, the displacement effect of irradiation on the devices is rarely reported, which can create defects to affect the electrical properties of the devices. Proton irradiation is one of the main particles in space environment, and will make both the ionization effect and displacement effect. So it is required to study the effects of proton irradiation on nitrided MOS capacitor and MOSFET for space applications. The previous report of proton irradiation based on the lateral MOSFETs only concentrated on the proton irradiation effects on the μ_FE_ and V_TH_, in which μ_FE_ is improved and V_TH_ is decreased with increasing doses, but the relative mechanisms are still unclear [11].

Therefore, in this paper the effects of 5 MeV proton irradiation on nitrided SiO_2_/4H-SiC MOS capacitors are studied in detail and the related mechanisms are revealed. With the analysis of the XPS measurement and annealing response of the samples, the ionization effect and displacement effect of proton irradiation on interface traps and near interface traps are clarified, and the mechanisms of the variation of the μ_FE_ are understood. By the experiments of the samples with different nitrogen content under same irradiation dose, the nitrogen passivation induced the shift of V_FB_ under irradiation is revealed. Finally, the gate oxide integrity, free carrier concentration (n_D_) in epilayer and positive bias stress stability of V_FB_ are also discussed, which are important for the power MOSFET.

## 2. Experiments

### 2.1. Test Devices

**Figure 1:**
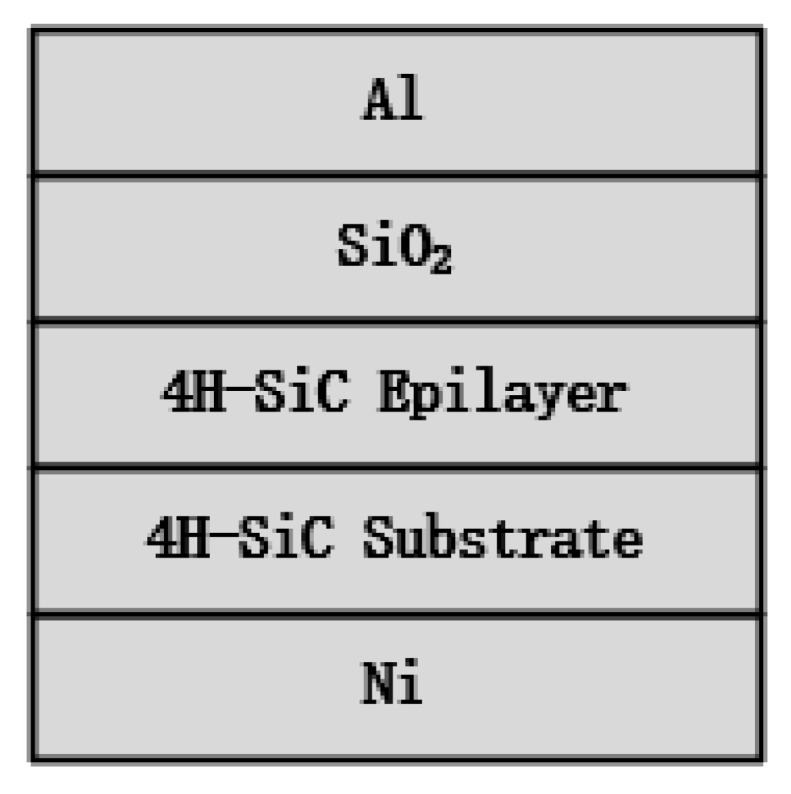
Schematic diagram of the nitrided SiO_2_/4H-SiC metal–oxide–semiconductor (MOS) capacitors.

The MOS capacitors under test were fabricated on a 4H-SiC epilayer with thickness of 5 μm and nitrogen-doped concentration of 1.77 × 10^16^ cm^−3^, grown on a 4° off-axis N^+^ type 4H-SiC substrate with thickness of 350 μm. Following the RCA cleaning, an HF dip, water rinsing, and thermal oxidation were carried out in a dry O_2_ environment at a temperature of 1300 °C. Afterwards, post oxidation NO annealing was performed under the condition of 1175 °C for 2 h. The thickness of the gate oxide is about 35 nm measured by spectroscopy ellipsometry (SE). Finally, nickel was evaporated for the backside ohmic contact and aluminum was sputtered on the oxide surface to form the gate electrode. The diameter of the gate electrode is 50 μm. In order to analyze the mechanisms of the shift of V_FB_ under proton irradiation, the samples were fabricated with changing annealing temperature and time, which are respectively 1100 °C with 1 h and 2 h, and 1250 °C with 1 h and 2 h. Schematic diagram of the nitrided SiO_2_/4H-SiC MOS capacitor is shown in Figure 1. The current to voltage (I-V) characteristics and HF (100 kHz) capacitance-voltage (C-V) measurements were measured on a CASCADE probe station using a Keysight B1505A semiconductor parameter analyzer at room temperature prior to the irradiation.

### 2.2. High Energy Proton Irradiation

**Figure 2:**
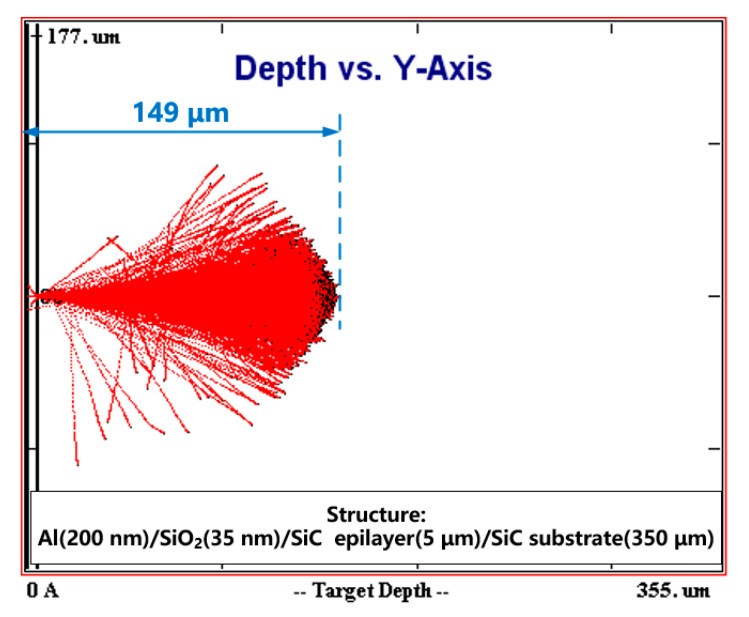
SRIM simulation of the MOS Capacitors injected by a 5MeV Proton beam.

Unbiased devices were irradiated with 5 MeV protons using the EN2 × 6 serial electrostatic accelerator (High Voltage Engineering Europa B.V., Amersfoort, Netherlands). The vertical irradiation to surface of the samples was performed in vacuum atmosphere at room temperature with the proton fluences of 5 × 10^12^ p/cm^2^, 2 × 10^13^ p/cm^2^, 5 × 10^13^ p/cm^2^, 1 × 10^14^ p/cm^2^, and 5 × 10^14^ p/cm^2^ under the same proton fluence rate of 1.39 × 10^10^ p/(cm^2^∙s), respectively. The proton energy was chosen from the energy range of protons in space environment, which is similar to the previous research on the irradiation effects of protons on 4H-SiC epilayers and MOSFETs [11,12]. The maximum incident depth is approximately 149 μm located in the substrate, which is determined by the Monte Carlo simulations using the SRIM software, as shown in Figure 2 [13]. According to the SRIM simulation of the ionization energy loss and the number of target vacancies versus the depth, the ionization effect and displacement effect of proton irradiation occur along the path of the injected protons during the proton irradiation. In order to analyze the mechanisms of the shift of V_FB_ under proton irradiation, the samples with different nitrogen contents were irradiated with the same dose of 2 × 10^13^ p/cm^2^. The I-V characteristics and C-V characteristics of these devices were re-measured at room temperature 24 h after each proton irradiation. The measurements of positive bias stress stability of V_FB_ were applied by positive bias stress of 3 MV/cm for the stress time in the range of 0–600 s, followed by HF (100 kHz) C-V measurements.

### 2.3. XPS Measurement and Annealing Experiment

XPS measurements were performed using a Thermo Scientific XPS (ESCALAB 250Xi) system, equipped with an Al Kα radiation source (1486.6 eV) for the excitation of photoelectrons. Argon ion sputtering was carried out in the same ultrahigh-vacuum chamber to remove the SiO_2_ layer gradually. The isochronal annealing experiments were performed in air ambient without gate bias at temperatures from 50 °C to 150 °C with each step of 50 °C [14]. The samples were held in 1 h, and then sequentially processed for total 3 h at the respective temperatures before cooling back down to room temperature for C-V characteristics measurements.

## 3. Results and Discussion

### 3.1. Gate Oxide Integrity

**Figure 3:**
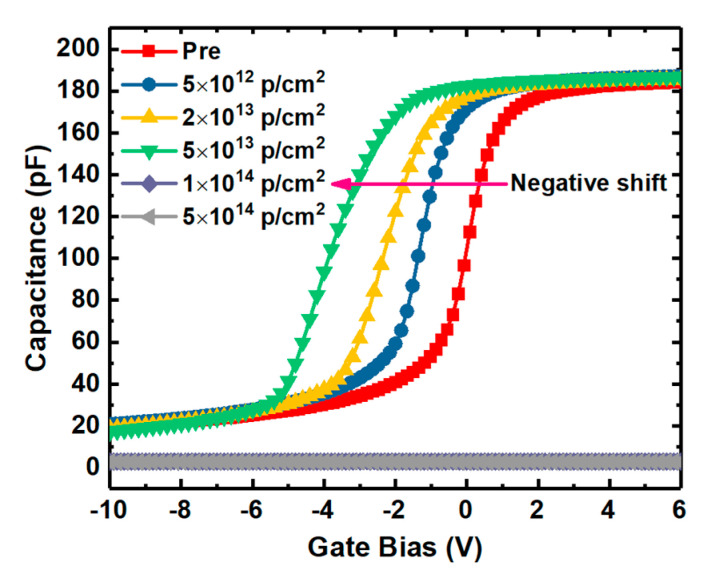
High frequency (HF) C-V characteristics of the samples irradiated by proton doses.

**Figure 4:**
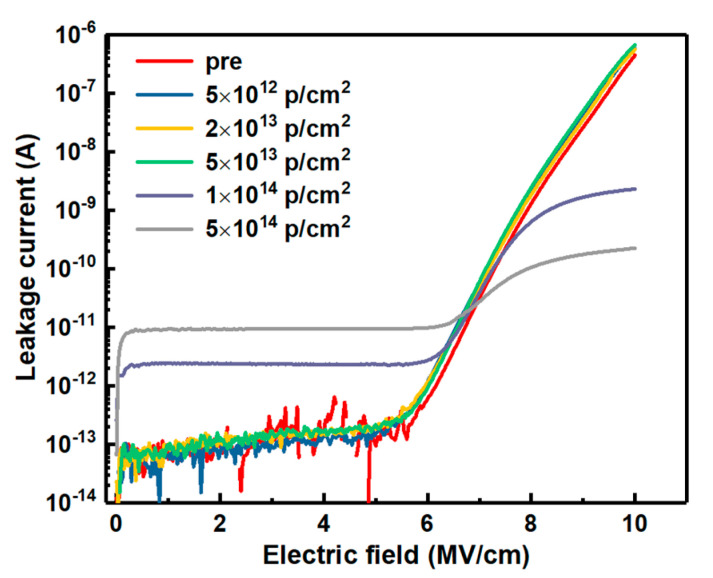
Typical current versus electric field of the samples with different doses of proton irradiation.

The HF C-V characteristics of the samples irradiated by proton doses are shown in Figure 3, in which the gate voltage was swept from depletion to accumulation. It can be seen that the samples were failed in the C-V characteristics when the irradiation dose is up to 1 × 10^14^ p/cm^2^. To evaluate the irradiation effect on the gate oxide integrity, the forward I-V characteristics were measured at room temperature by sweeping the gate voltage up to 35 V. The forward current versus electric field (I-E) of the samples with different doses of proton irradiation is shown in Figure 4, which is transformed from the measured I-V characteristics. It can be observed that the leakage current of the samples irradiated by the dose up to 5 × 10^13^ p/cm^2^ remains very little value as the electric field is smaller than 6 MV/cm, resulted from the direct tunneling of the oxide layer, and rapidly increases because of Fowler-Nordheim (F-N) tunneling, complied with F-N rule as the electric field higher than 6 MV/cm. But the leakage current of the samples irradiated by the dose over 5 × 10^13^ p/cm^2^ is increased obviously with the increasing irradiated doses in the low electric field and not complied with F-N rule in the high electric field, which could be owing to the damage of the barrier between SiO_2_ and 4H-SiC caused by the defects in the oxide layer generated by the displacement effect of proton irradiation, and this is also the reason lost C-V characteristic for the samples after irradiation dose of 1 × 10^14^ p/cm^2^. Therefore, this could lead to the failure of the MOSFET due to losing gate control.

### 3.2. Free Carrier Concentration

**Figure 5:**
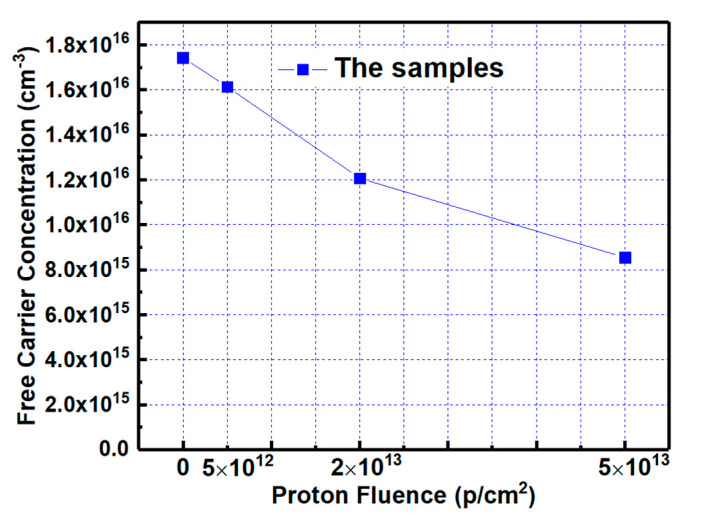
The free carrier concentration of the epilayer versus proton fluence.

The n_D_ in the epilayer versus proton fluence is extracted by HF C-V characteristics of the samples, as shown in Figure 5. It can be seen that the n_D_ is reduced with increasing irradiation dose. The previous experiment showed that the displacement effect of the proton irradiation introduces several electron traps and their energy levels are located at 0.18 eV, 0.2 eV, 0.4 eV, 0.72 eV, 0.76 eV, and 1.09 eV below the conduction-band edge, respectively [12], which measured on the 6.5 MeV proton-irradiated 4H-SiC epilayer using deep-level transient spectroscopy (DLTS), which is almost of similar energy and range of doses as our experiment. The concentrations of the defects are linearly related to irradiation dose. These electron traps capture free carrier, resulting in the n_D_ reduced as carrier removal effect. This effect could significantly influence the ON-state resistance of the power MOSFET, finally leading to the failure of the device by worsening of the resistivity of lightly doped drift region and the pinching off of JFET region.

### 3.3. Near Interface Traps

**Figure 6:**
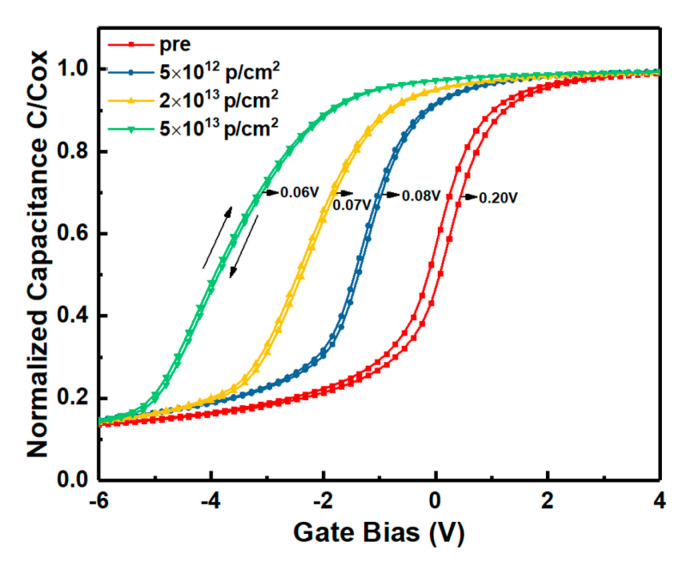
Bidirectional HF C-V characteristics of the samples irradiated by proton doses.

The normalized bidirectional HF C-V characteristics of the samples irradiated by proton doses are shown in Figure 6. In the measurements, the gate voltage was swept from depletion to accumulation, and then swept back to depletion. The hysteresis voltage (∆V) is related to the un-trapped NIETs which could trap electrons at the accumulation condition [15]. The areal density of un-trapped NIETs can be calculated by (Cox/qS)∙∆V, where Cox is oxide capacitance, q is electronic charge, S is area of oxide capacitance, as shown in Table 1. It can be observed that the number of un-trapped NIETs is decreased with the increasing irradiation dose. However, the number of un-trapped NIETs is affected by the number and the trapping state (trapped or un-trapped state) of the NIETs. The former can be influenced by the displacement effect of proton irradiation and the latter can be affected by the ionization effect of proton irradiation.

**Figure 7:**
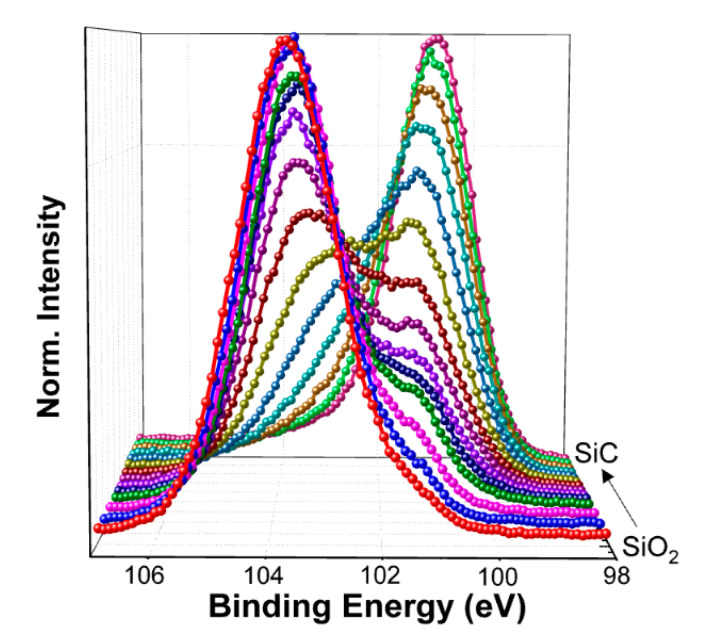
Si 2p core level X-rays photoelectron spectroscopy (XPS) spectra taken from the different depths of the transition of the sample without irradiation.

**Figure 8:**
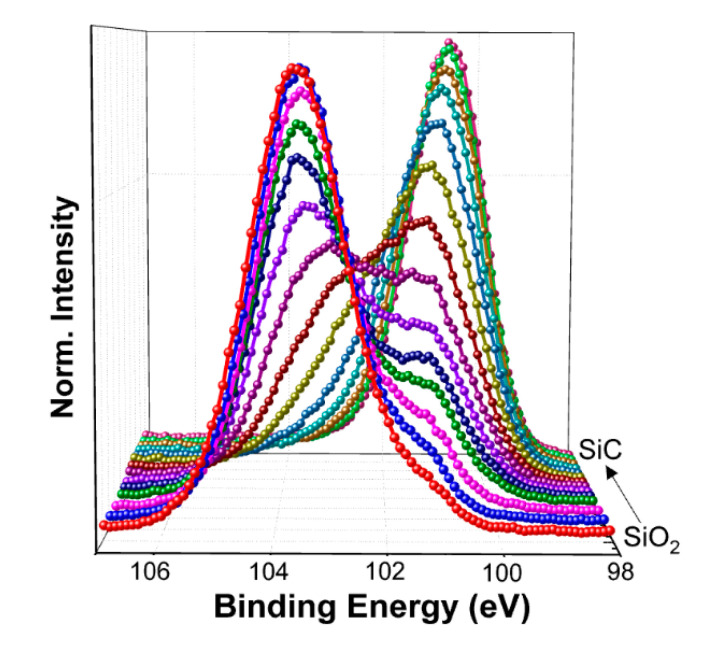
Si 2p core level XPS spectra taken from the different depths of the transition of the sample with the irradiation dose of 5 × 10^13^ p/cm^2^.

**Figure 9:**
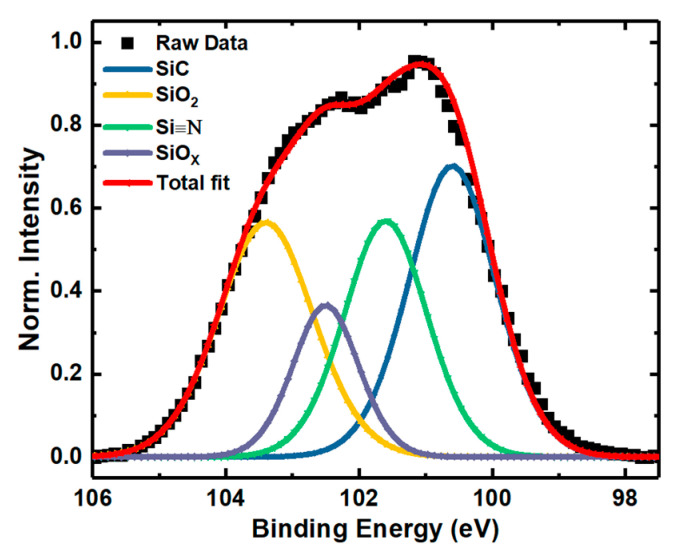
Si 2p core level XPS spectrum at the near interface of the samples without irradiation.

**Figure 10:**
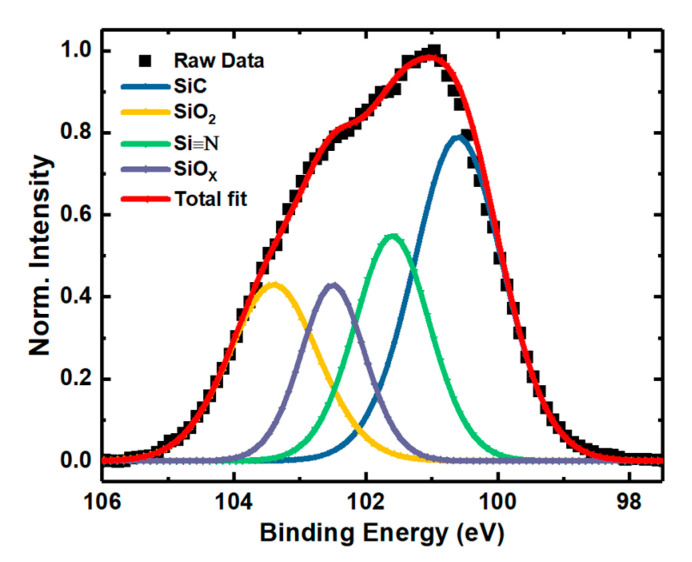
Si 2p core level XPS spectrum at the near interface of the samples with the irradiation dose of 5 × 10^13^ p/cm^2^.

**Figure 11:**
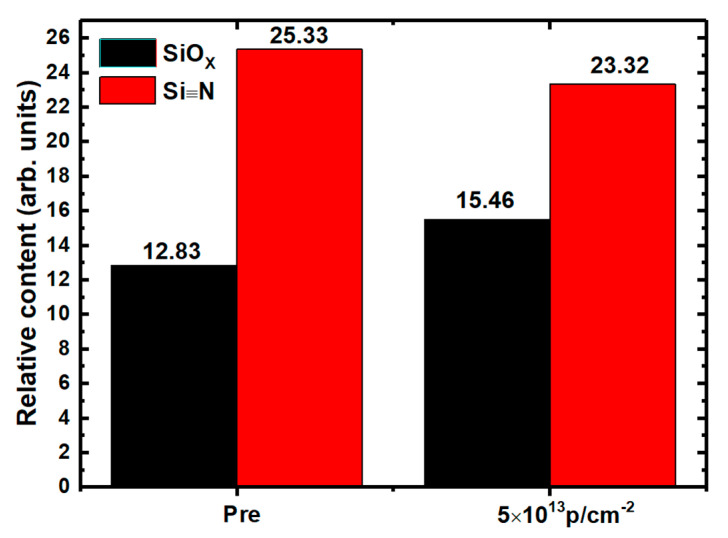
The relative contents of silicon suboxide and silicon nitride in the Si 2p core level spectra of the samples without irradiation and with the irradiation dose of 5 × 10^13^ p/cm^2^.

To analyze the displacement effect on NIETs, the XPS measurements of the samples without irradiation and with the irradiation dose of 5 × 10^13^ p/cm^2^ (the maximum irradiation dose before failure of the samples) were performed, in which argon ion sputtering was carried out to remove the SiO_2_ layer gradually and each step of sputtering time is 10 s (about 0.8 nm in depths). The XPS spectra of Si 2p core level taken from the different depths of the transition layers from SiO_2_ to SiC are shown in Figure 7 and Figure 8, respectively. From this process the XPS spectra at the near interface can be obtained and compared. The results of deconvolution of the Si 2p core level XPS spectra at the near interface for the samples are shown in Figure 9 and Figure 10, respectively. Before the curve fitting, Shirley background subtraction was performed and all spectra were charge compensated relative to the binding energy of the C-C bond (284.6 eV). Then XPS spectra were resolved as sums of several components taking each peak of a Gaussian distribution. Four main components are observed in the Si 2p spectra, which are Si-C, Si≡N, Si-O_X_, and SiO_2_ located at 100.6 eV, 101.6 eV, 102.5 eV, and 103.4 eV binding energies, respectively. The relative contents of the intermediate oxidation state of Si-O_X_ and Si≡N in the Si 2p core level spectra for the samples without irradiation and with irradiation dose of 5 × 10^13^ p/cm^2^ are shown in Figure 11, obtained by XPS analysis software (Thermo Avantage). It can be seen that the content of Si-O_X_ is increased slightly and Si≡N is decreased a little after irradiation. It can be considered that the breaking of Si≡N made by displacement effect of proton irradiation leads to more Si-O_X_, but the quantity is only 8%. Considering the NIET originates in Si-O_X_ and the NIHT originates in Si≡N [16,17,18], the number of both traps is not significantly changed by the displacement effect. The trapping state of the NIETs can be markedly changed by trapping electrons during irradiation generated by the ionization effect along the path of the injected proton. Therefore, the decrease of the number of un-trapped NIETs after irradiation is mainly affected by the ionization effect of the proton irradiation, which can be beneficial to the μ_FE_ of MOSFET [19,20,21].

### 3.4. Flat-Band Voltage

**Figure 12:**
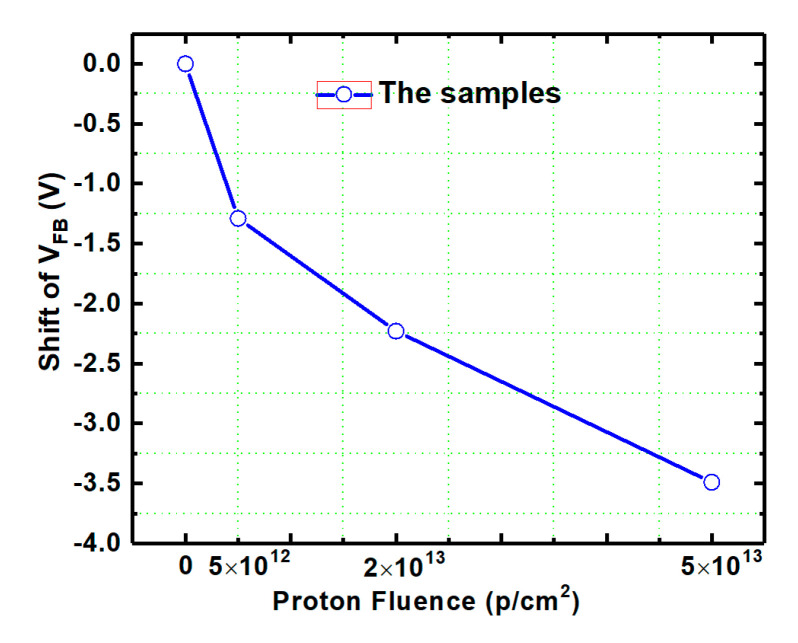
The shift of V_FB_ of the samples versus proton fluence extracted from the HF C-V characteristics.

**Figure 13:**
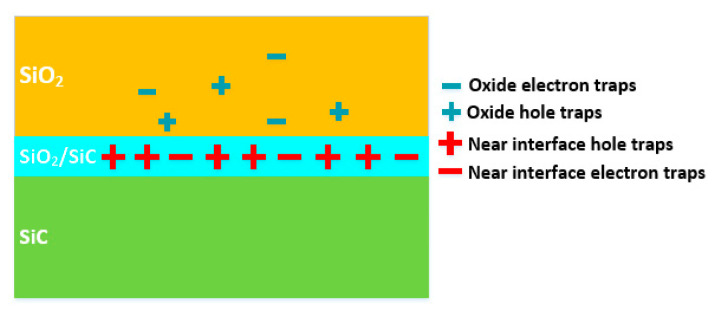
The schematic diagram of main traps affecting V_FB_ during irradiation nearby the SiO_2_/4H-SiC interface.

**Figure 14:**
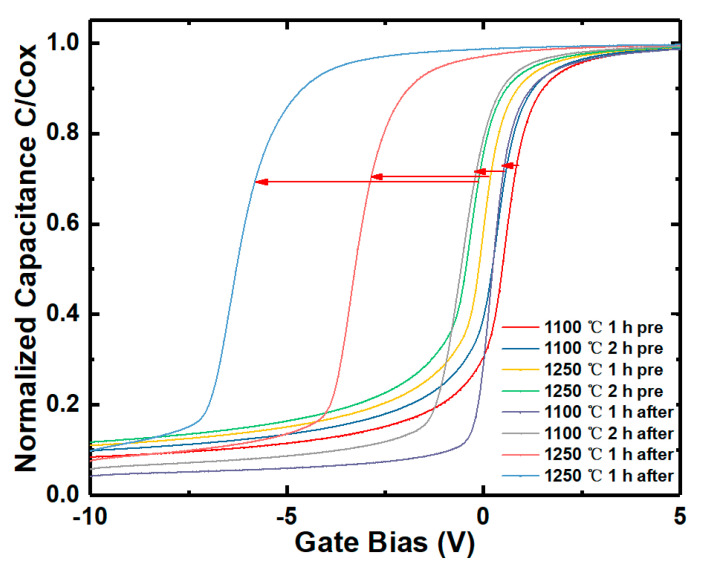
HF C-V characteristics of the samples with different nitriding process irradiated by same proton dose of 2 × 10^13^ p/cm^−2^.

The shift of V_FB_ of the samples compared with the un-irradiation one versus proton fluence extracted from the HF C-V characteristics of the irradiated samples (in Figure 3) is shown in Figure 12. Before failure, the V_FB_ of the samples decreases obviously with the increase of irradiation dose and the shift of V_FB_ is −3.49 V after the irradiation dose of 5 × 10^13^ p/cm^−2^, which means a significant increment of the net positive charges nearby the SiO_2_/4H-SiC interface during irradiation. The schematic diagram of main traps affecting V_FB_ during irradiation nearby the SiO_2_/4H-SiC interface is shown in Figure 13. As discussed from XPS spectra above, the number of NIETs and NIHTs are not significantly changed by the displacement effect during proton irradiation. Therefore, the effect of proton irradiation on V_FB_ is mainly affected by the ionization effect of the proton irradiation, depending on the dominate traps near the interface which could be the oxide hole traps formed by the oxygen vacancy or NIHT induced by nitrogen passivation. So the samples with different nitrogen content varied by adjusting the NO annealing time were performed under same irradiation dose of 2 × 10^13^ p/cm^−2^. The annealing time is respectively 1 h and 2 h at temperatures of 1100 °C and 1250 °C. HF C-V characteristics of the samples with different nitrogen content irradiated by same proton dose is shown in Figure 14. Considering the different thickness of oxide (tox) of the samples, the extracted variation of the areal density of effective dielectric charge (∆Nef) of the samples is shown in Table 2. It can be seen that the ∆Nef of the samples with annealing time of 2 h are obviously more than that with 1 h under both temperatures of 1100 °C and 1250 °C. It means that the ∆Nef is significantly related to the nitrogen content. The nitrogen passivation not only reduces the numbers of the NIETs but also generates a large number of the NIHTs, leading to the dominant hole traps nearby the SiO_2_/4H-SiC interface. When the protons penetrate though the devices the ionization effect of proton irradiation creates electron-hole pairs along the path of the injected proton. The dominant NIHTs capture more holes in this event, resulting in the generation of net positive charges nearby the SiO_2_/4H-SiC interface. This is the reason for the larger negative shift of V_FB_ after proton irradiation. This serious problem must be addressed because it could induce the MOSFETs to not turn off normally. Therefore, it is necessary for the nitrogen content controlling to carefully choose annealing condition to balance the improvement of μ_FE_ induced by the decrease of NIETs and the shift of V_TH_ caused by the formation of NIHTs for the application of MOSFETs in ionization irradiation environment.

### 3.5. Interface Traps

**Figure 15:**
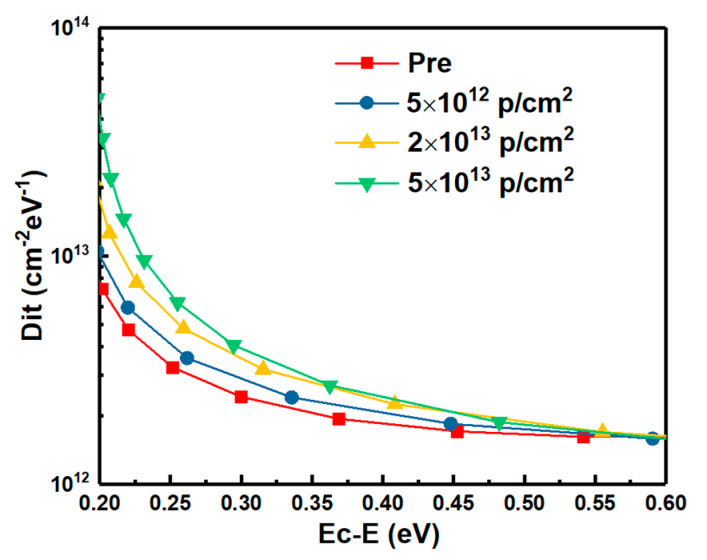
Energy distributions of interface states densities of the samples irradiated by proton doses obtained by Terman’s method.

**Figure 16:**
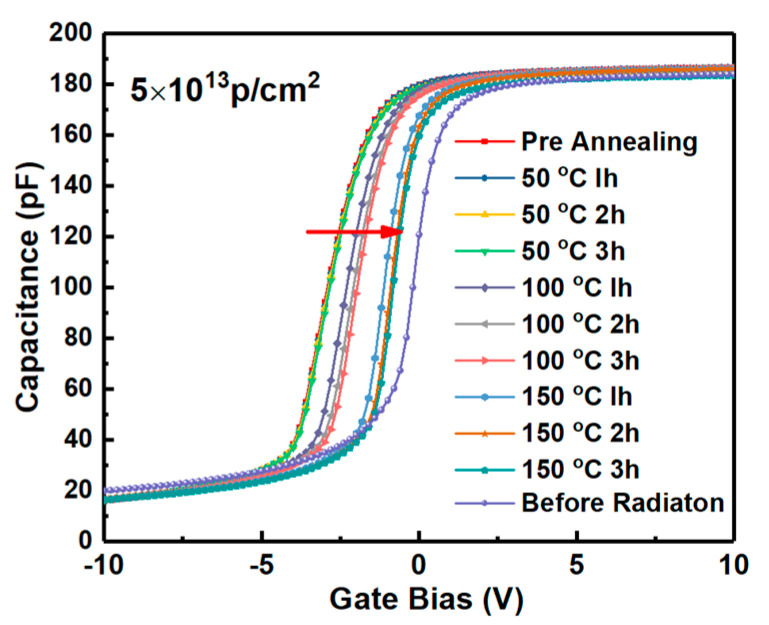
HF C-V characteristics of the samples at different stages of the isochronal annealing.

**Figure 17:**
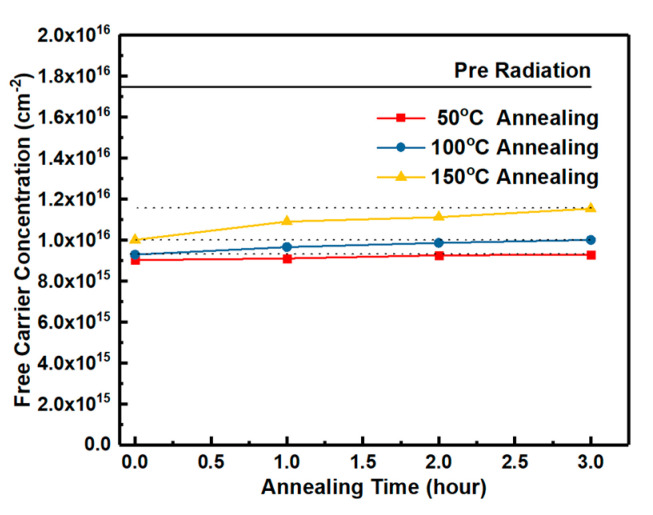
The extracted free carrier concentration of the epilayer at different stages of the isochronal annealing.

**Figure 18:**
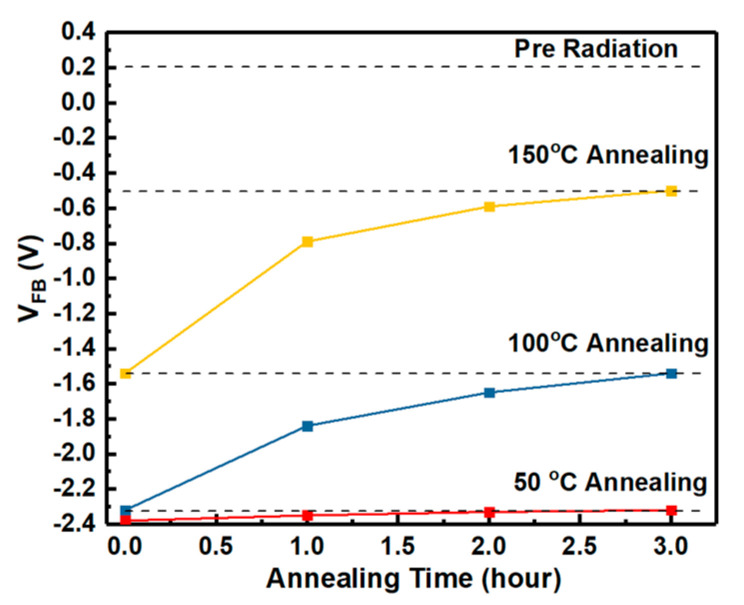
The extracted flat-band voltage of the sample at different stages of the isochronal annealing.

**Figure 19:**
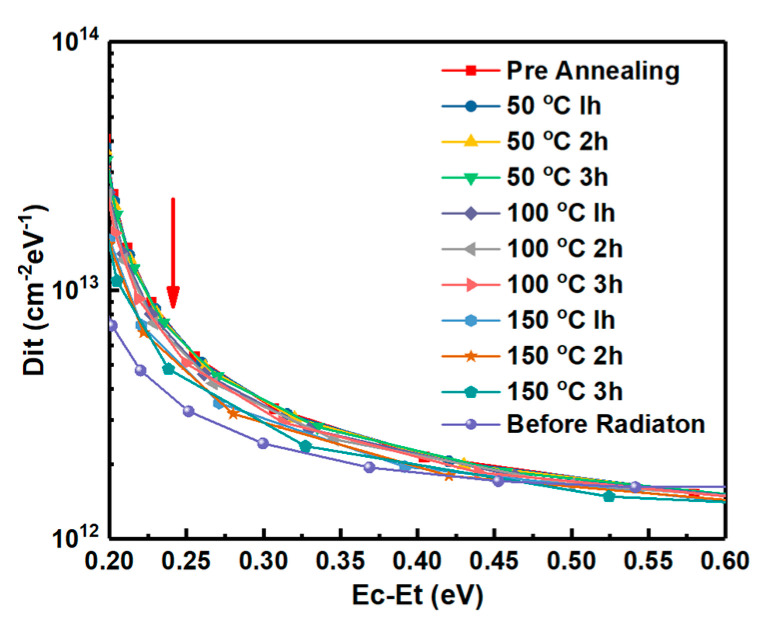
Energy distributions of interface states density in the sample at different stages of the isochronal annealing.

Energy distributions of interface states densities of the samples irradiated by proton doses obtained by Terman’s method based on measured HF C-V characteristics (in Figure 3) are shown in Figure 15. It can be observed that the Dit is increased with the increasing irradiation dose. The displacement effect of the proton irradiation causes various types broken bonds between atoms, when the protons penetrate though the interface. The defects generated at the interface caused by the displacement effect could be the reason for the increase of Dit. But the worse of Dit is present in the irradiation of only ionization effect [3]. Which effect has more significant influence on Dit is analyzed by the annealing response of the samples without irradiation and with the irradiation dose of 5 × 10^13^ p/cm^2^. The HF C-V characteristics of the sample at different stages of the isochronal annealing are shown in Figure 16. The relationship between the n_D_ and annealing condition is shown in Figure 17. It can be seen that the n_D_ only returned to 29% of pre-irradiation levels after the isochronal annealing. The extracted V_FB_ of the sample at different stages of the isochronal annealing are showed in Figure 18. It can be noticed that the V_FB_ of the samples returned to 70% of pre-irradiation levels after the isochronal annealing. Energy distributions of Dit of the sample at different stages of the isochronal annealing are plotted in Figure 19. It can be seen that the recovery of Dit of the sample is 32% which is similar to the recovery of n_D_ (29%) mainly affected by the displacement effect and different from the recovery of V_FB_ (70%) mainly influenced by ionization effect, so it can be considered that the worse of Dit is mainly affected by the displacement effect of proton irradiation.

**Figure 20:**
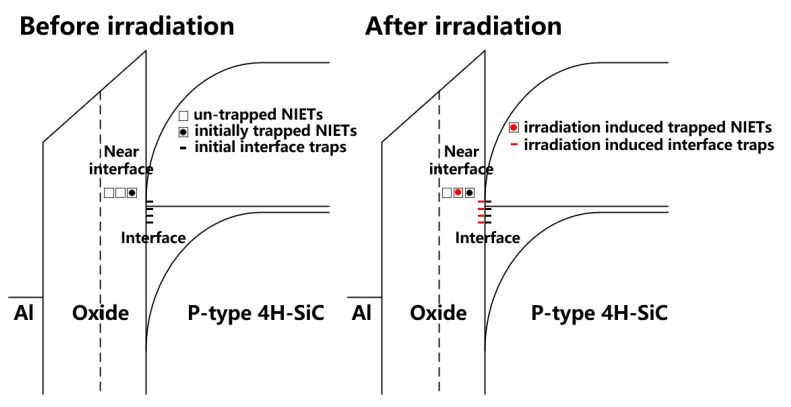
Energy band diagrams for the effect of proton irradiation on NIETs and interface traps.

Up to now, the irradiation effect on NIETs and interface traps have been analyzed and it can be summarized in Figure 20. The μ_FE_ of MOSFET is affected by the interface traps located close to the conduction band and the NIETs near the conduction band, and the effect of the latter one is more significant [19,20,21]. The electrons in the inversion layer can directly tunnel or tunnel via interface traps to the un-trapped NIETs when the MOSFET devices are biased into strong inversion, which reduce the number of channel conducting electrons resulting in the effective reduction of μ_FE_. This could explain the results of previous study, in which the μ_FE_ of MOSFET irradiated by 5 MeV proton is increased with the increasing irradiation dose [11]. In this case, the reduction of the un-trapped NIETs by the ionization effect of proton irradiation could be the reason for the improvement of μ_FE_ with increasing irradiation doses.

### 3.6. The Positive Bias Stress Stability of V_FB_

**Figure 21:**
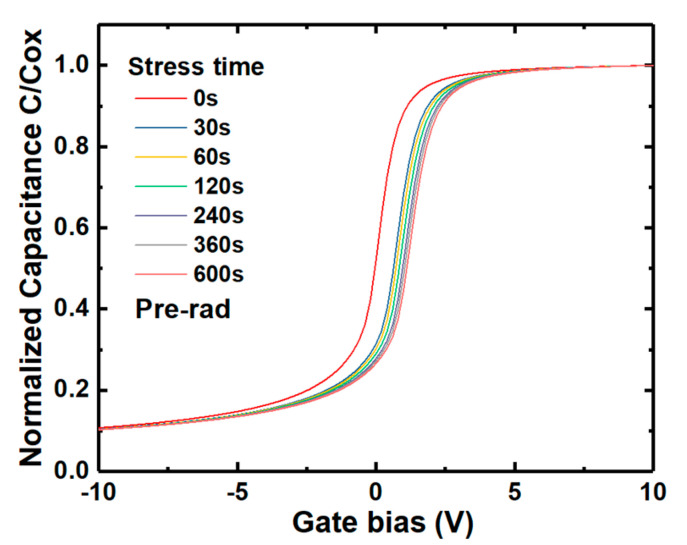
HF C-V characteristics of the un-irradiated samples with the stress time.

**Figure 22:**
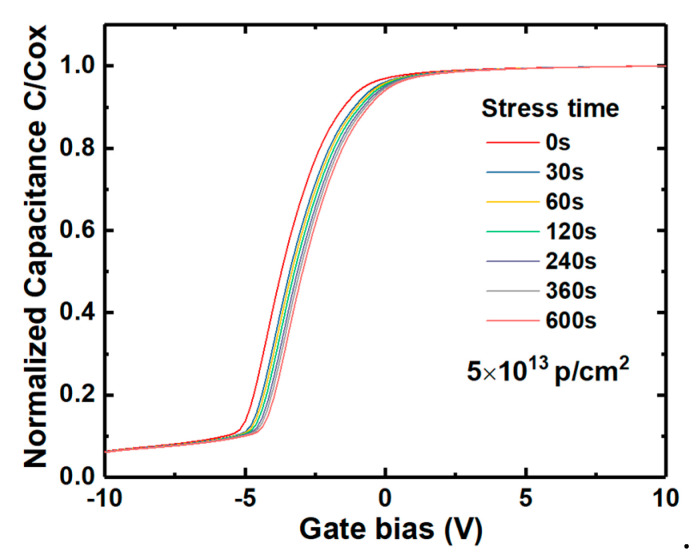
HF C-V characteristics of the samples irradiated by the dose of 5 × 10^13^ p/cm^2^ with the stress time.

**Figure 23:**
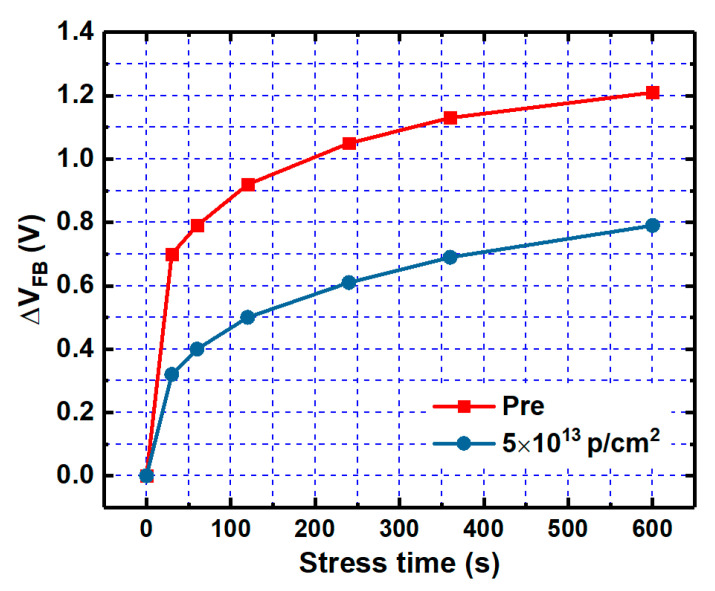
The variation of V_FB_ of the samples without irradiation and with the irradiation dose of 5 × 10^13^ p/cm^2^ versus the stress time.

Electrons tunneling into the NIETs far away from the interface and oxide electron traps are hardly released quickly when suffering time-dependent bias stress (TDBS), resulting in the problem of the threshold voltage stability [22]. It has been confirmed that n-channel MOSFET operated in strong inversion (in a positive gate bias) can be equivalent to an n-type MOS capacitor in the accumulation state. Thus, it is necessary to investigate the irradiation effect on the positive bias stress stability of V_FB._ The samples without irradiation and with the irradiation dose of 5 × 10^13^ p/cm^2^ were applied by positive bias stress of 3 MV/cm for different stress time in the range of 0–600 s, followed by HF C-V measurements, as shown in Figure 21 and Figure 22, respectively. The variation of V_FB_ (∆V_FB_) compared with the samples before the positive bias stress extracted from the C-V measurements of the samples versus the stress time is shown in Figure 23. Compared with the un-irradiated samples, the ∆V_FB_ of the samples after irradiation is decreased after every stress time, which could be attributed to the NIETs far away from the interface and oxide electron traps filled by the electrons resulted from the ionization effect of proton irradiation. The decrease of ∆V_FB_ after irradiation could contribute to the threshold voltage stability of n-channel MOSFETs.

Up to now, the ionization effect on the electrical characteristics can be summarized as that traps present at the regions from SiO_2_ to interface (shown in Figure 13) can be filled during irradiation caused by ionization effect, which could be beneficial to the μ_FE_ and threshold voltage stability of n-channel MOSFETs, but cause serious problem of obvious negative shift of V_FB_ and V_TH_.

## 4. Conclusions

In this paper the effects of 5 MeV proton irradiation on nitrided SiO_2_/4H-SiC MOS capacitors are studied in detail and the related mechanisms are revealed. Because of the displacement effect of proton irradiation in the oxide, the gate oxide integrity is broken after the irradiation dose of 5 × 10^13^ p/cm^2^, which could lead to the failure of power MOSFET. The Dit is increased with the irradiation doses, and the annealing response suggests that the worse of Dit is mainly caused by displacement effect of proton irradiation. However, the XPS measurement shows that the quantity proportion of breaking of Si≡N induced by displacement is only 8%, which means that the numbers of NIETs and NIHTs are not significantly changed by displacement effect. The measurements of bidirectional HF C-V characteristics and positive bias stress stability show that the number of un-trapped NIETs and oxide electron traps decreased with increasing irradiation doses because they are filled by electrons resulted from the ionization effect of proton irradiation, benefiting to the μ_FE_ and threshold voltage stability of MOSFETs. The obviously negative shift of V_FB_ resulted from the dominant NIHTs induced by nitrogen passivation capture more holes produced by ionization effect, which has been revealed by the experimental samples with different nitrogen content under same irradiation dose. This serious problem must be addressed because it could induce the MOSFETs to be not turned off normally. Therefore, it is necessary for nitrogen content controlling to carefully choose annealing condition to balance the improvement of μ_FE_ induced by the decrease of both of NIETs and the shift of V_TH_ caused by the formation of NIHTs for the application of MOSFETs in ionization irradiation environment.

## Figures and Tables

**Table 1:** Extracted hysteresis voltage and the areal density of un-trapped NIETs of the samples irradiated by proton doses.

Irradiation Dose (p/cm^2^)	Hysteresis Voltage (V)	Areal Density of un-Trapped NIETs (cm^−2^)
pre	0.20	1.2 × 10^11^
5 × 10^12^	0.08	4.7 × 10^10^
2 × 10^13^	0.07	4.2 × 10^10^
5 × 10^13^	0.06	3.6 × 10^10^

**Table 2:** Extracted the shift of V_FB_ and the variation of areal density of effective dielectric charge of the samples with different nitriding process irradiated by the same proton dose of 2 × 10^13^ p/cm^−2^.

Annealing Condition	the Shift of V_FB_ (V)	Tox (nm)	∆Nef (cm^−2^)
1100 °C 1 h	0.3	48.0	1.4 × 10^11^
1100 °C 2 h	0.9	48.8	4.0 × 10^11^
1250 °C 1 h	3.2	52.8	1.3 × 10^12^
1250 °C 2 h	5.9	56.8	2.2 × 10^12^
